# A cohort study protocol to analyze the predisposing factors to common chronic non-communicable diseases in rural areas: Fasa Cohort Study

**DOI:** 10.1186/s12889-016-3760-z

**Published:** 2016-10-18

**Authors:** Mojtaba Farjam, Hossein Bahrami, Ehsan Bahramali, Javad Jamshidi, Alireza Askari, Habibollah Zakeri, Reza Homayounfar, Hossein Poustchi, Reza Malekzadeh

**Affiliations:** 1 Noncommunicable Diseases Research Center, Fasa University of Medical Sciences, Fasa, Iran; 2 Department of Medical Pharmacology, Fasa University of Medical Sciences, Fasa, Iran; 3 Division of Cardiovascular Medicine, Department of Medicine, Keck School of Medicine, University of Southern California, Los Angeles, USA; 4 Department of Preventive Medicine, Keck School of Medicine, University of Southern California, Los Angeles, USA; 5 Cardiology Department, Fasa University of Medical Sciences, Fasa, Iran; 6 Department of Medical Genetics, Fasa University of Medical Sciences, Fasa, Iran; 7 Anesthesiology Department, Fasa University of Medical Sciences, Fasa, Iran; 8 Digestive Diseases Research Institute, Tehran University of Medical Sciences, Tehran, Iran

**Keywords:** Non-communicable diseases, Cohort study, Risk factors, Developing countries

## Abstract

**Background:**

Non-communicable diseases (NCDs) have become the main causes of morbidity and mortality even in rural areas of many developing countries, including Iran. In view of this increased risk, Fasa Cohort Study (FACS) has been established to assess the risk factors for NCDs with the ultimate goal of providing optimal risk calculators for Iranian population and finding grounds for interventions at the population level.

**Methods:**

In a population-based cohort, at least 10,000 people within the age range of 35 to 70 years old from Sheshdeh, the suburb of Fasa city and its 24 satellite villages are being recruited. A detailed demographic, socioeconomic, anthropometric, nutrition, and medical history is obtained for each individual besides limited physical examinations and determination of physical activity and sleep patterns supplemented by body composition and electrocardiographic records. Routine laboratory assessments are done and a comprehensive biobank is compiled for future biological investigations. All data are stored online using a dedicated software.

**Discussion:**

FACS enrolls the individuals from rural and little township areas to evaluate the health conditions and analyze the risk factors pertinent to major NCDs. This study will provide an evidence-based background for further national and international policies in preventive medicine. Yearly follow ups are designed to assess the health events in the participating population. It is believed that the results would construct a contemporary knowledge of Iranian high risk health characteristics and behaviors as well as the platform for further interventions of risk reduction in a typical Iranian population. Constantly probing for future advances in NCDs prevention and management, the accumulated database and biobank serves as a potential for state of the art research and international collaborations.

## Background

Iranian population is estimated to be more than 80,000,000 based on 2011 national census [1]. A few decades ago, the main causes of mortality in Iran, like most other developing countries, were communicable diseases [2]. After operating the WHO recommendations regarding disease control and by practicing prevention via Iranian model of health network in rural and urban areas, most communicable diseases were successfully controlled and the mortality due to these diseases dramatically decreased [3]. Health interventions in rural health houses and full-coverage vaccination were the two effective preventive programs which largely contributed to this success. However, as expected, the non-communicable diseases (NCDs) became more prevalent, and indeed, collectively they became the main causes of morbidity and mortality in the Iranian society such that their share in all-cause mortality increase from 57 % in 1990 to 76 % in 2010 [4]. These changes are similar to the trends observed in many developing countries [5, 6].

It is generally believed that the reason for the emergence of NCDs as the leading causes of morbidity and mortality, is the change in people lifestyle [7]. Due to modernization of the Iranian society, diet has been changed not only in the urban regions, but also in the rural community [7]. Moreover, successful infectious disease control and promotion of life standards has elongated the life span and as a result, the number of people vulnerable to non-communicable diseases including cardiovascular, metabolic, renal and malignant diseases has increased. With the alarmingly high prevalence of NCDs in Iran [8], applying preventing strategies are of paramount importance. Prevention against NCDs is not achievable by any single interventions. This is in contrast to communicable diseases, and although research on the development of vaccines for some NCDs are going on [9–12], currently it is not a readily available option worldwide. Till emergence of any paradigm shift in NCDs, preventive measures as vaccines, different levels of NCDs prevention should be defined in an evidence-based manner which apparently demands systematic and reliable data acquisition [13–16].

Ischemic heart disease, stroke, and other vascular diseases, collectively considered as cardiovascular diseases (CVD) encompass the major cause of death followed by cancer [17]. Regarding CVDs compared to other diseases, they have a handful of modifiable risk factors. Although longitudinal studies to search for these modifiable risk factors date back to more than half a century ago in developed countries, the discoveries achieved do not completely address the developing world’s current health problems in that these countries are not totally an equal match to the developed societies. This is particularly important in view of our previous work in a smaller cohort that showed that the prevalence of common risk factors for CVD in Iranian population is at high as American population [18]. Undoubtedly, baseline characteristics of the population studied, determine the risk estimates thus achieving to a precise and accurate prognostic measure obligates a longitudinal survey in the population of interest. Furthermore, the somewhat unique aspects of the Iranian lifestyle as one of the few ancient civilizations remained as a single country today, as well as the ethnic diversities and considerable genetic heterogeneity in this part of the world necessitate a separate approach to look for the gene-environment interactions as potential CV risk determinants.

According to the American Heart Association (AHA) recommendations, coronary heart disease risk among adults of 40 years of age and more without previous CVD history ought to be determined every 5 years [19]. Conventional risk factors used in risk calculators though proved to be accurate and reliable in different populations, are not ideal in minor ethnicities and disease states like diabetes. Attempts are made as well to incorporate other novel risk factors into the calculators of risk to yield in a better risk stratification tool. There are considerations at the same time about the imposed financial burden of the components of the risk calculators related to the costly laboratory investigations for developing countries. While still more justifications are needed to apply the AHA recommendations in developing countries, a recent study, has focused on the recalibration of the Framingham office-based CV risk calculator and reported that the measures obtained from history, physical examinations and anthropometric indexes can be used comparably to yield in similar risk estimates at the lowest cost compared to the available accepted laboratory based risk calculators [20].

Regarding the mentioned shortcomings with the available tools for determination of CVDs risk in Iran, looking for more readily available means taking into account the ethnic diversities, limited financial resources and the advantages of an established primary health care network seems mandatory. Cohort studies are of special importance to analyze the risk factors of predisposition to NCDs. This will also significantly contribute to designing preventive strategies by the policy makers. The cohort data can be used in organization of almost all levels of disease prevention and evidence-based real-time algorithm tailoring in both preventive and clinical issues [16, 21]. Fasa University of Medical Sciences (FUMS), an institute dedicated to improving the health standards, [22] has planned to build up a population-based cohort study in its territory to acquire an image of the NCDs prevalence and incidence and at the same time research on the related risk factors. This is under the supervision of the deputy for research and technology of the Iranian ministry of health (MOH).

## Methods/design

### Participant recruitment

Fasa cohort study (FACS) has been designed to evaluate the risk factors pertinent to predisposing the inhabitants of the rural region of Fasa to NCDs including the most common ones. The study is a population-based longitudinal survey with a total follow- up span of 15 years. People are invited to participate in the study by the rural health care worker (Behvarz), the representatives of the primary health care system in every health house in the villages and small towns [23]. Behvarz knows every single individual invited and their health status having registered all villagers of his or her territory in the first place. In Iranian model of the health network, Behvarz is of extraordinary importance as she/he have been selected from the habitants of same towns and villages and have underwent special training programs before they started as the health care worker in the primary health care network. They cover a population of around 200–2000 population and are in direct contact with people of their region with routine and programmed follow ups and recordings of basic health status of their region. Behvarz follow ups are held in the health houses where she regularly visits people and performs basic physical examinations such as blood pressure control as well as health events recordings. Behvarzes served as the inviters to the FACS because they were familiar with every habitant of the region and people trust them. They have been briefed about the study protocol and goals in special training sessions by the PIs of the study. Upon a well-rehearsed method, Behvarzes counted and invited every eligible habitant of the region in a clockwise manner eventually selecting every living place from the health house.

### Target region and target population

Fasa city is located in the eastern part of the province of Fars, in southwest of Iran with a population of about 250,000. A rural region known as Sheshdeh (28°56′56.0″N 53°59′26.9″E) with a total population of 41,000 was chosen for the cohort study. People within the age range of 35–70 years (11,097 individuals) were planned to participate as the target population in the cohort since they are old enough that have been exposed to health threads and risk factors and young enough not to develop CV events and NCDs endpoints. With the planned follow up time of 15 years they’re expected to experience study outcome variables with the highest possible rate.

**Fig. 1:**
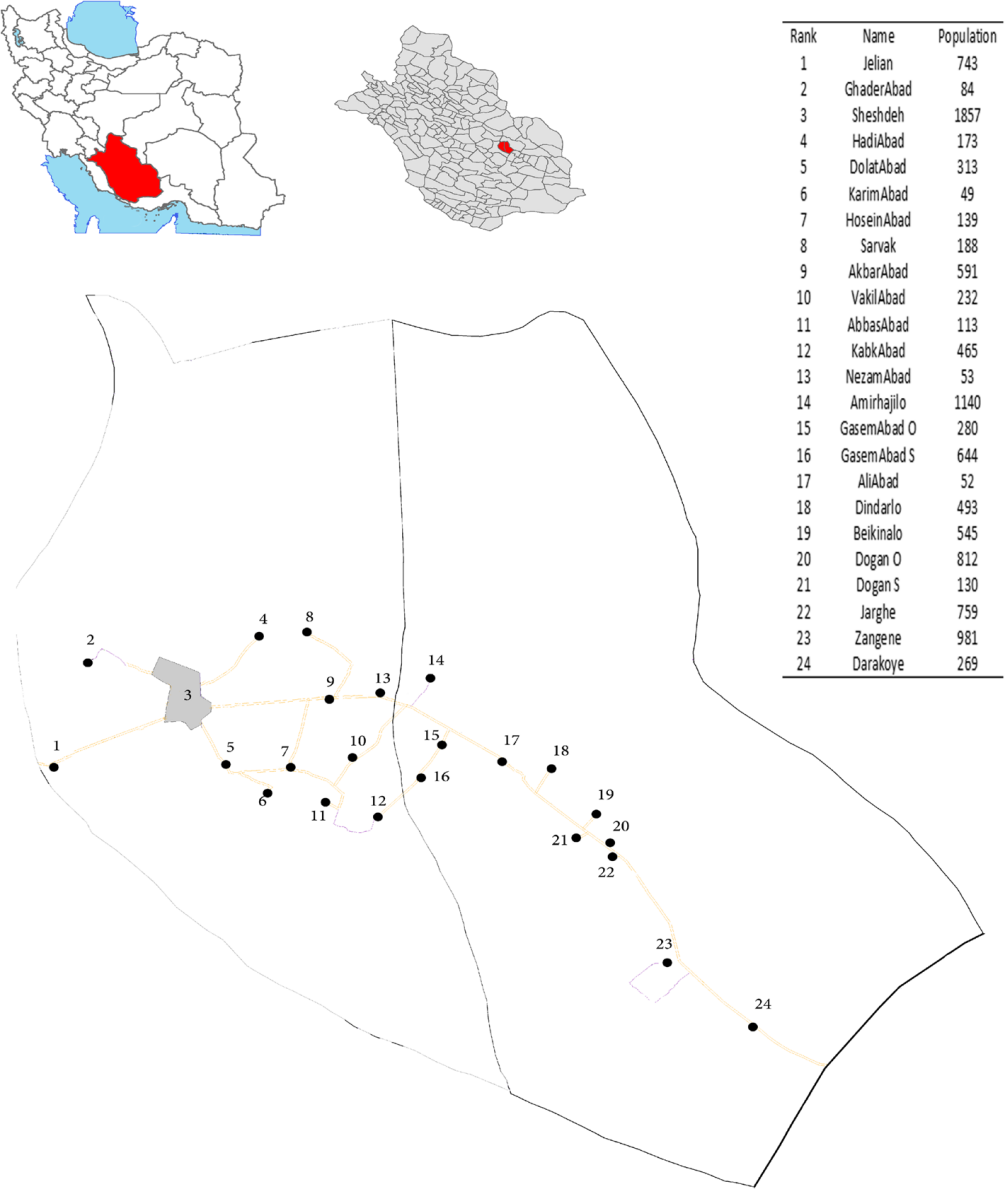
The location and the name of Fasa Cohort Study districts and their population of 35–70 years old

The ethnicities of people living in this region is diverse, including Fars, Turks and Arabs. They live in a small town (Sheshdeh) and 24 surrounding villages. The name of these districts and their population of 35–70 years old are represented in Fig. 1.

### The field office

An office in the field was funded in the main town of the region. The building has one reception desk and 12 rooms for samplings, physical examinations and interviews. Waiting rooms are equipped with display monitors that constantly guide people through different steps of enrollment process. All essential equipment including computers, sampling facilities and physical exam devices are provided in the field.

### The laboratory

The laboratory is located in the NCD research center central laboratory at FUMS. State of the art laboratory facilities are available for processing the samples. A biobank with five −70 °C refrigerators and UPS system to control the electricity equipped with real time temperature and electricity controller has been provided.

### Human resources

A field team works daily in the field office. The team consists of a field supervisor, two physicians, interviewers, nurses, sampling technicians, driver and office boy. Field supervisor is in charge for surveillance of the ongoing interviews and monitoring that data acquisition is carried out according to the protocols.

All personnel have been selected from a number of eligible volunteers after a primary interview and upon a completed training course in the MOH. All of them have been approved through detailed tests after training workshops. The interviewers have been practically trained to ask questions from the participants using digital data acquisition sheets in addition to valid and standard questionnaires and screening tools. They are divided into general, medical and nutrition interviewers based on the questionnaire type they administer. They have academic degrees from bachelor to master related to their job. Four Laboratory technicians and a lab supervisor work in the laboratory. The laboratory personnel have been selected by exams after training about the cohort. They are familiar with the details of coding process, laboratory issues and biobank management and work under direct supervision of laboratory supervisor. The supervisor is responsible for laboratory coding, recording and biobank organization. All lab people are familiar with the cohort protocols and have academic degrees in the field of medical laboratory sciences.

A scientific team of principal investigators (PIs) including cardiologist, nutritionist, pharmacologist, epidemiologist, geneticist and anesthesiologist specialized in pain management supervises the cohort working both in the field and the headquarters. All PIs are academic investigators affiliated to Fasa, Shiraz and Tehran universities of medical sciences and have been updated regarding the cohort study. They are responsible for all executive and scientific processes. They supervise thfe process of data gathering and laboratory function, observe the administrative issues related to the project and data cleaning and perform the quality control procedures of the data. The scientific analysis of the variables is done by this team regularly. The main PI is the corresponding manager who chooses the field supervisor.

### Work steps in the field office

**Fig. 2:**
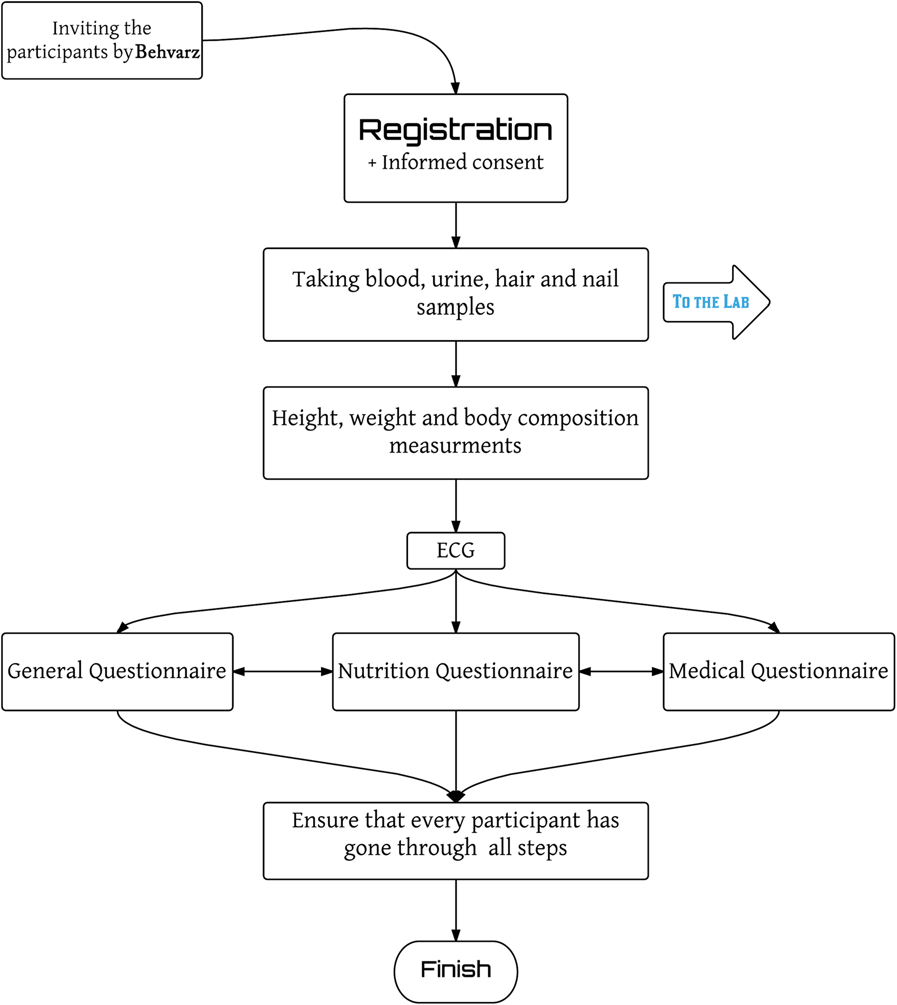
the schematic presentation of registration process in the field

A schematic presentation is provided in Fig. 2.

#### Step 1- Registration

The registration process is based on national IDID code of each individual. The field supervisor registers participants by checking their ID cards and a unique number is attributed to each one (PCID) and from then on, it will be available on all questionnaires, forms and sample tubes. One of the interviewers also helps field supervisor with the registration. In all steps of the cohort study, PCID is searchable. A cohort ID card will be issued at the same time, with the digital bar code, photo and the attributed number. A registry is also performed in a separate notebook. The registered individual should be checked against the previously prepared list of the invited participants by the Behvarz. For every participant, an informed consent letter is filled before registration. All the questionnaires are electronic, using a dedicated online software. We used the advantage of electronic questionnaires for improving the accuracy and validity of the entered data. They were designed to be smart in a way that potentially inaccurate data, pre specified outliers or invalid variable types are highlighted and the data entry doesn’t proceed unless the registrar rechecks and accepts the entry.

#### Step 2- Anthropometrics measurement

In this step, weight, height, and an estimation of body water is evaluated by using Bioelectrical impedance analysis (tanita BC-418, tanita corp, japan) for every participant.

#### Step 3- Laboratory sampling

For this cohort, the collection and saving of samples are of extreme importance. Invitees have been asked not to trim or color their hair and nails for 2–4 weeks and maintain fasting state for 10–14 h prior to referral. Blood, urine, hair and nail samples are taken from all the participants. The sampling is done by professional sampling personnel using modern tools. Samples are kept in codified tubes and containers.

A total of 25 ml of blood is taken, comprising one 7-ml clot tube and three 6-ml EDTA tubes. About 15 ml of urine is taken from every participant. Nail and hair are taken too. At least 1 mm of all nails of hand and foot carried in aluminum foil is kept in zip keeps containing humid absorber. For hair samples, occipital hair is taken from the hair root and put in zip keep bags in which humidity absorber is included.

#### Step 4- Electrocardiography

All participants have 12-lead electrocardiograms (EKG) recorded before the interviews. They are asked to shave their precordium at the time of invitation for the best possible result attained. EKGs are all stored in the standard format (Health Level-7) and a dedicated software (Cardiax®) automatically makes diagnoses and provides electronic data exports to be incorporated into the central data acquisition software. Automatic diagnoses will be reviewed by cardiologists further upon the related research protocols per needed. Totally 156 parameters are recorded for each participant per EKG which includes 12-lead deflection voltage amplitudes, durations, intervals and ST segment deviations as well as heart rate and vector analyses. Data quality is assessed each month after interpretations by the steering committee.

#### Step 5- Interviews to fill the questionnaires

##### General interview

In this section, the general characteristics of the participants are recorded. The general questionnaire is subdivided to nine sections comprising personal information, general information, socioeconomic status, occupational status, home location and type of fuel used, lifestyle, anthropometric measurements, mobile use, and pesticide use. In each subsection the detailed information for every participant is recorded (details of the questionnaire are available at ncdrc.fums.ac.ir/collaboration).

##### Medical interview

Data acquisition forms are filled in asking about any previous medical history of every participant. Validated screening questionnaires are utilized as well to obtain data on the incident common NCDs, dental health, physical activity, sleep quality and duration, fertility, substance and alcohol abuse and smoking. The full length data gathering sheet and questionnaire is available at ncdrc.fums.ac.ir. Participants are also asked to bring their medications at the time of interview to register the drug history with maximum precision. History of prior hospitalizations are recorded and a positive family history for NCDs are sought.

##### Nutrition interview

The modified food frequency questionnaire (FFQ) is administered in this phase to evaluate the eating habits and foods consumed by the participants. The FFQ is a semi-quantitative 125-item inventory. The inventory is used to obtain information on dietary intake over a 1-year period and is a Willett format questionnaire [24] modification based on Iranian food items. It includes a list of foods (with standard serving sizes) commonly consumed by Iranians. Individuals are requested to report their frequency of consumption of a given serving of each food item during the past year, on a daily, weekly, monthly or yearly basis. A standard portion size is designated for each item by using United States Department of Agriculture (USDA) serving sizes (e.g. bread, one slice; dairy, one cup).

#### Step 6- Physical exam

Five nurses are in charge for taking resting heart rate and blood pressure, examining the oral hygiene, inspecting for alopecia and/or hirsutism, analyzing the participants’ posture and looking for the gross abnormalities in limbs and torso. They are trained to measure and record the blood pressure in both arms consecutively. After 15 min repeated measurements are done and recorded. Heart rate is recorded in sitting position 15 min apart as well. Initially all the nurses were granted with a certificate after completing the training courses and every 3 months, they have received repeated instruction for certificate renewal.

### Laboratory work in the lab

After taking samples from participants in the field, they are transferred in cool boxes to the laboratory for processing. Different laboratory data are provided for each participant. These include complete blood count (CBC); blood biochemical parameters measurements (fasting blood sugar (FBS), serum total cholesterol (TC), triglyceride (TG), high density lipoprotein (HDL) and low density lipoprotein (LDL) cholesterol, alanine aminotransferase (ALT), asparagine aminotransferase (AST), alkaline phosphatase, gamma glutamyl transpeptidase, urea, and creatinine levels and urine analysis (color, specific gravity, appearance, PH, nitrite, bilirubin, urobilinogen, protein, glucose, and blood). The laboratory data are recorded in an electronic database. These data then are imported to the main database including every participant detailed information which were recorded through the questionnaires.

### Sample saving

For blood specimen saving, 1.4 ml 2D crayotubes are used. There are smart 12-digit code on every tube which can be read by smart scanner. Twelve cryotubes are prepared as represented in the Table 1.

**Table 1:** Saved samples during the study for every individual

Sample	Number of cryotubes	Volume (ml)
Whole blood	2	1
Plasma	6	1
Buffy coat	2	1
Serum	2	1

The cryotubes are stored in −70 refrigerators. The samples are put with a specific order in the box codified for every individual participant.

### The follow-up phase

This study includes a 15-year follow- up phase. As one of the important pitfalls of follow-up phase could be lack of knowledge in staffs, every individual who is in charge of follow up will be educated for understanding their role before the follow up starts. The follow up data will be recorded in a software which is designed especially for this purpose.

The follow up team comprises a physician/nurse, interviewers (which are preferably nurses) and a laboratory technician. They are responsible for yearly follow up and work under the supervision of the main PI and the field director. An outcome review team including three internists along with follow-up team will determine the final diagnosis or the cause of any death of participants. At the time of enrolment every participant signs an informed consent in order to let the follow-up team to access their past and future medical records.

There are two types of follow-up, passive and active. In passive follow-up the data will be gathered through self-reports and reports of diseases and cancer registry centers (and also other centers related to health services such as: private clinics, general physicians, forensics centers, medical insurance, laboratories). The deaths will be recorded and if an outcome of interest (such as cardiovascular diseases and cancer) was confirmed in a participant, the participants will be invited to the laboratory and blood sample will be taken from them. Besides getting data via passive reports, most cases will be followed-up actively using phone interviews by the physician or the interviewers in yearly intervals. If there was no answer in a phone call after six times in three different days of 3 weeks, the follow-up team will go to the postal address of the participant and interview will be done face to face. Behvarzes will facilitate this process. The deaths will be recorded and in case of outcome of interest, the participants will be invited to the laboratory and blood sample will be taken from them.

To investigate changes in risk or protective factors of diseases, in 5, 10 and 15th years of follow-up (2nd, 3rd and 4th Screenings), the re-sampling (blood, urine, hair and nail) will be carried out and new lifestyle questionnaire and FFQ will be filled out for all alive participants.

### Quality control

Surveillance systems are of crucial importance and the quality control team at the initial enrollment phase has a practically validated checklist to clearly address a variety of quality measures of data collection. The team supervisor is an epidemiologist who is not one of the investigators of FACS. They cover all three aspects of the data and specimen acquisition process: general questionnaires and anthropometric measurements, medical and disease screening questionnaires, and biologic samples acquisition and biobank maintenance. Furthermore, the control will be operated every 3 months by an external reviewer who is familiar with the entire process of registration at FACS. If any draw backs observed, they would be taken into consideration for the following clean up phase. The quality control administrator who is one of the PIs has full access to the database and codified information. In case of any need for clean up another team member retrieves the codified data for the cleanup.

The FACS software is designed to make basic checks automatically as well. Data fields are sensitive to outliers which has been defined by the investigators and the software alarms the operators to confirm the values they are entering before the final registration. Inaccurate data in terms of variable types, length and measurement levels are also identified and the final approval depends on an extra step of PI validation.

## Discussion

There are gaps between research-based evidence, health policies and clinical practice in developing countries [25]. Incorporating the evidence provided by studies in developing countries into policy making is a critical step in improving the health status in these countries [26, 27]. Acknowledging that, FACS was founded by NCD research center to contribute to tailoring Iranian evidence-based health and medical policies. By this view, and by the support of the MOH, some major management barriers such as the limitations in financial and human resources [28] were overcome. Technical issues were solved by means of several expert sessions at the MOH and using the opinions of international collaborators. The health deputy in Fasa also played a special role in offering its network facilities for recruitment of the people.

One problem might be the immigration of the participants. To address that we engaged actively in the development of a nationwide health indexes registration system (SIB network) which actively records every Iranian’s health status by means of an online software. The software allows tracking of people in the health network and facilitates the outcome measurements for FACS. The two disease registration systems available in FUMS as population-based registry for myocardial infarction (FaRMI) and hospital-based registry for systolic heart failure (FaRSH) are customized as well to notify the investigators of FACS in case of any incident outcomes for FACS participants. Death registers are also reviewed weekly by the follow up team and are checked against the national IDs of participants registered in FACS.

To further support national and international collaborations, the data will be available to investigators who accept terms and conditions noted in the official website of the FACS (ncdrc.fums.ac.ir). The laboratory will continue functioning in later phases and the biobank will be operating and ready to be used for researchers and collaborators who like to investigate in the field after authorization by the steering committee.

## Abbreviations

AHA: American heart association
ALT: Alanine aminotransferase
AST: Asparagine aminotransferase
CBC: Complete blood count
CVD: Cardiovascular diseases
EDTA: Ethylenediaminetetraacetic acid
EKG: Electrocardiograms
FACS: Fasa cohort study
FBS: Fasting blood sugar
FFQ: Food frequency questionnaire
FUMS: Fasa University of Medical Sciences
HDL: High density lipoprotein
LDL: Low density lipoprotein
MOH: Ministry of health
NCD: Non-communicable diseases
PCID: Persian cohort ID
PIs: Principal investigators
TC: Serum total cholesterol
TG: Triglyceride
USDA: United States department of agriculture
WHO: World health organization
